# A workflow task force affects emergency physician compliance for point-of-care ultrasound documentation and billing

**DOI:** 10.1186/s13089-016-0041-0

**Published:** 2016-05-20

**Authors:** Resa E. Lewiss, Jessica Cook, Allison Sauler, Nicholas Avitabile, Nicole L. Kaban, Jeffrey Rabrich, Turandot Saul, Sebastian D. Siadecki, Dan Wiener

**Affiliations:** Department of emergency medicine, Department of Radiology, University of Colorado Hospital, Aurora, CO USA; Department of emergency medicine, Yale-New Haven Hospital, New Haven, CT USA; Department of emergency medicine, Mount Sinai St. Luke’s Mount Sinai West, New York, NY USA; Department of emergency medicine, St. Barnabas Hospital, Bronx, NY USA; Department of emergency medicine, Mount Sinai Beth Israel Hospital , New York, NY USA; Bronx-Lebanon Hospital Center, Bronx, NY USA

**Keywords:** Point-of-care ultrasound, Workflow, Ultrasound workflow, Documentation compliance

## Abstract

**Background:**

Emergency point-of-care ultrasound (POC u/s) is an example of a health information technology that improves patient care and time to correct diagnosis. POC u/s examinations should be documented, as they comprise an integral component of physician decision making. Incomplete documentation prevents coding, billing and physician group compensation for ultrasound-guided procedures and patient care. We aimed to assess the effect of directed education and personal feedback through a task force driven initiative to increase the number of POC u/s examinations documented and transferred to medical coders by emergency medicine physicians.

**Methods:**

Three months before a chosen go-live date, departmental leadership, the ultrasound division, and residents formed a task force. Barriers to documentation were identified through brain storming and email solicitation. The total number and application-specific POC u/s examinations performed and transferred to the healthcare record and medical coders were compared for the pre- and post-task force intervention periods. Chi square analysis was used to determine the difference between the number of POC u/s examinations reported before and after the intervention.

**Results:**

A total of 1652 POC u/s examinations were reported during the study period. Successful reporting to the patient care chart and medical coders increased from 41 % pre-task force intervention to 63 % post-intervention (*p* value 0.000). The number of scans performed during the 3-month periods (pre-intervetion, post-intervention 0–3 months, post-intervention 3–6 months) was similar (521, 594 and 537). When analyzed by specific application, the majority showed a statistically significant increase in the percentage of examinations reported, including those most critical for patient care decision making: (EFAST (41 vs. 64 %), vascular access (26 vs. 61 %), and cardiac (43 vs. 72 %); and those most commonly performed: biliary (44 vs. 61 %) and pelvic (60 vs. 66 %). Of the POC u/s studies coded and reported for reimbursement, 15.9 % were billed before intervention and 32 % were billed after intervention (*p* value: 0.000).

**Conclusions:**

The formation of a workflow solution task force positively affected emergency physician compliance with POC u/s documentation for coding and billing over a 6-month period. Further investigation should assess the long-term effect of the intervention and whether this translates into increased revenue to the department.

## Background

Health information technology is increasingly recognized as an essential tool for improving patient safety and quality of care. Emergency point-of-care ultrasound (POC u/s) is an example of a healthcare technology that improves patient care and decreases the time to making a correct diagnosis [1–4]. POC u/s examinations should be appropriately documented and the resultant emergency health records (EHRs) should accurately reflect the level of patient care provided [5]. Incomplete documentation prevents coding, billing and physician compensation for patient care provided and ultrasound-guided procedures performed [6]. Prior research suggests that changing the behavior of medical providers requires active intervention based on assessment of existing barriers to change. Also, multiple simultaneous interventions are more likely to be effective than any individual intervention alone [7]. The aim of this proof-of-concept study was to evaluate if POC u/s documentation by emergency medicine attending physicians improved after the formation of a task force and the implementation of multiple specific interventional initiatives.

## Methods

Study design, population and setting: This was a retrospective quality improvement project. Administrative and residency leadership faculty, two emergency ultrasound fellows, an emergency medicine resident from each class, and the lead medical coder for the emergency department formed a 13-member task force. The group met monthly for 3 months prior to a go-live date of January 1, 2014. The group recognized that the number of POC u/s examinations being performed for patient care was greater than those documented in the EHR and even greater than those subsequently coded for billing and reimbursement. Through brain storming at monthly meetings and via electronic mail communications, the task force defined and explored the suspected barriers, and sought appropriate solutions and interventions.

Based upon the barriers, the task force prioritized those for which there could timely and sustainable solutions (Table 1).

**Table 1:** Point-of-care ultrasound workflow barriers and interventions

Barriers	Interventions and solutions
Scan completed but documentation incomplete	Real-time transfer of study to image archiving software
	Direct transfer of Qpath™ report to EHR
	Attending physicians encouraged to transfer studies in real time
Q-path™ worksheet not completed real time	Emphasis on scan and documentation as a single event
Machinery upkeep	“Image of the week” email
	Consultative services, e.g. general surgery and obstetrics–gynecology educated regarding proper care of equipment
Wireless transmission delays	Biomedical engineering correspondence
	Information technology correspondence
	Hardware vendor correspondence
Q-path logout time too short	Workstation logout time increased
Provider awareness of workflow process	Faculty meeting presentation
	Resident conference presentation
	Educational module distributed to all staff
	Educational module placed on centralized website for immediate access and review
	Laminated reminder signs placed at physician workstations
Attending compliance	Daily reports generated listing attending physicians with studies performed but not documented or vice versa
	Regular follow-up from department chairman with noncompliant attending physicians

The task force met monthly and progressively introduced methods of individual and group education and personalized feedback to encourage a behavioral change in documentation compliance. Leading up to the go-live date, both resident and attending physicians received in-person and online instruction on the workflow process. A tutorial was posted online for continual review through a centralized website. Other interventions included mass electronic mail messaging, advertising of the task force initiative, and placing reminder signs on all computers at both sites to complete documentation. Emphasis was placed on performance of the ultrasound scan and documentation as one event. Daily electronic reports were generated. These listed individual attending physicians who performed an ultrasound but failed to document in the EHR or conversely, those who documented in the EHR that an ultrasound examination had been performed, but did not submit the ultrasound report itself to the medical coders for billing. The department chairman conducted individual follow-ups with the attending physicians identified in these daily reports. The task force identified physician education as the primary focus for improvement. Items for reinforcement included the definition of a patient care (diagnostic) scan, the specific steps to be completed in the workflow process, and the departmental ultrasound billing capabilities.

The evaluation was performed over a 9-month period (October 2013–June 2014) at an academic institution with a 3-year emergency medicine residency and an emergency POC u/s fellowship. We analyzed the existing data 3 months pre-intervention (01 October 2013 through 31 December 2013). Data collection began 01 January 2014. We then analyzed the data 3 and 6 months post-intervention. Patient data were initially known to the task force investigators involved, and then de-identified. There was no record of patient name, medical record number, or demographics included in the study. There was no funding support for the project. This study was determined exempt from institutional review board approval.

### Data and analysis

Data were collected for the 3 months before and at 3 and 6 months after the study go live intervention date. SAS^®^ 9.3 software was utilized. Chi square analysis was used to determine the difference between the total number of POC u/s examinations correctly performed and documented pre- and post-task force intervention. Application-specific data pre- and post-intervention were also analyzed.

## Results

**Fig. 1:**
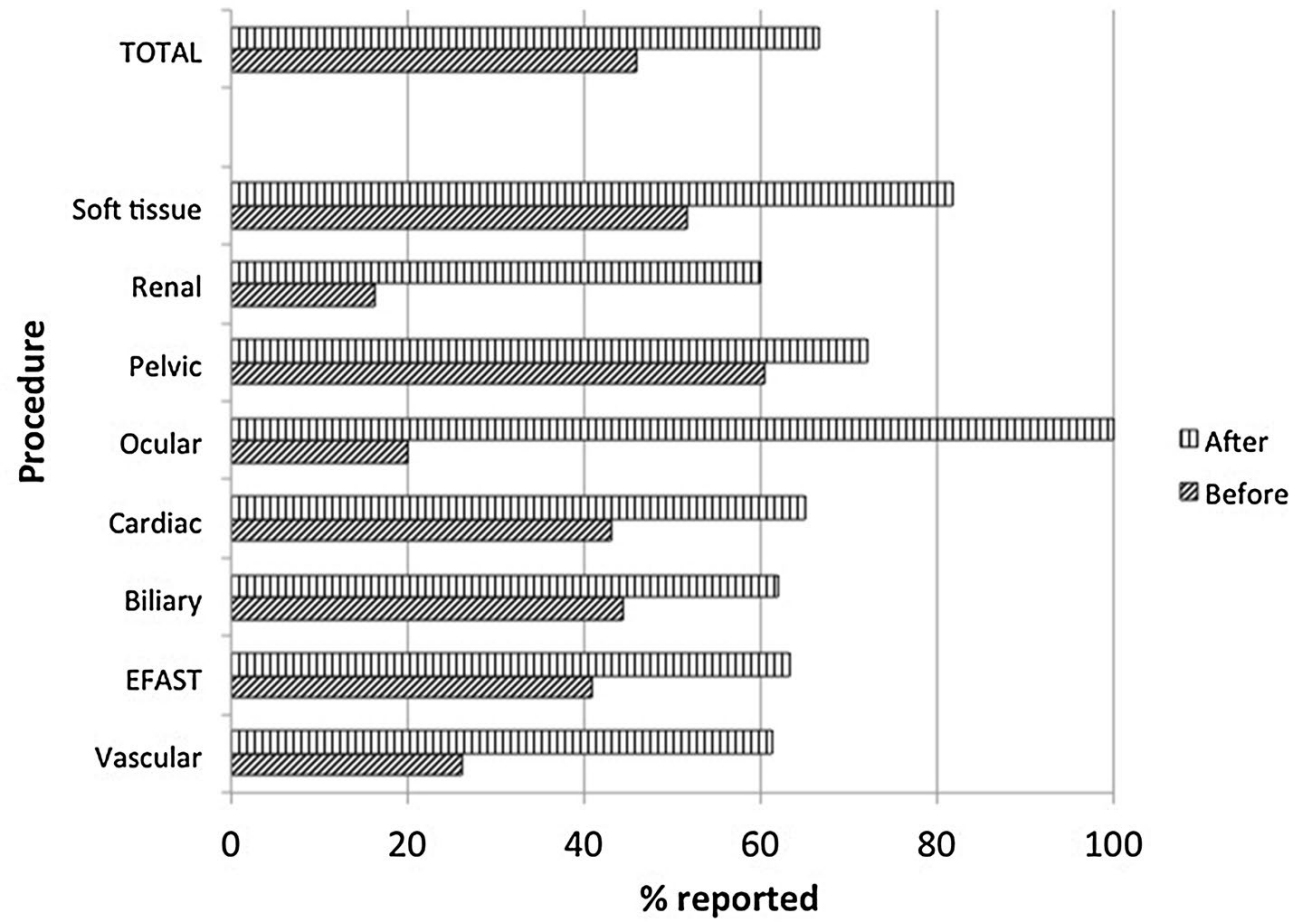
The percentage of scans reported to the medical coders in the pre- and post-intervention periods. *X-axis* represents the percentage of POC u/s scans documented and reported for coding (*slanted dark hashed bar* pre-intervention data; *vertical hashed bar* post-intervention data). *Y-axis* identifies the number of application specific and number of total POC u/s examinations

1652 total POC u/s scans were reported during the study period. The number of scans performed during the three consecutive 3-month periods pre- and post-intervention was similar (521, 594 and 537). Successful documentation increased from 41 % pre- to 63 % post-intervention (*p* value 0.000). When analyzed by specific application, the majority of POC u/s examinations showed a statistically significant increase in the percentage of scans documented. This included those most commonly performed on critical care patients: (EFAST (41 vs. 64 %), vascular access (26 vs. 61 %), and cardiac (43 vs. 72 %); and those most commonly performed: biliary (44 vs. 61 %) and pelvic (60 vs. 66 %) (Fig. 1).

**Fig. 2:**
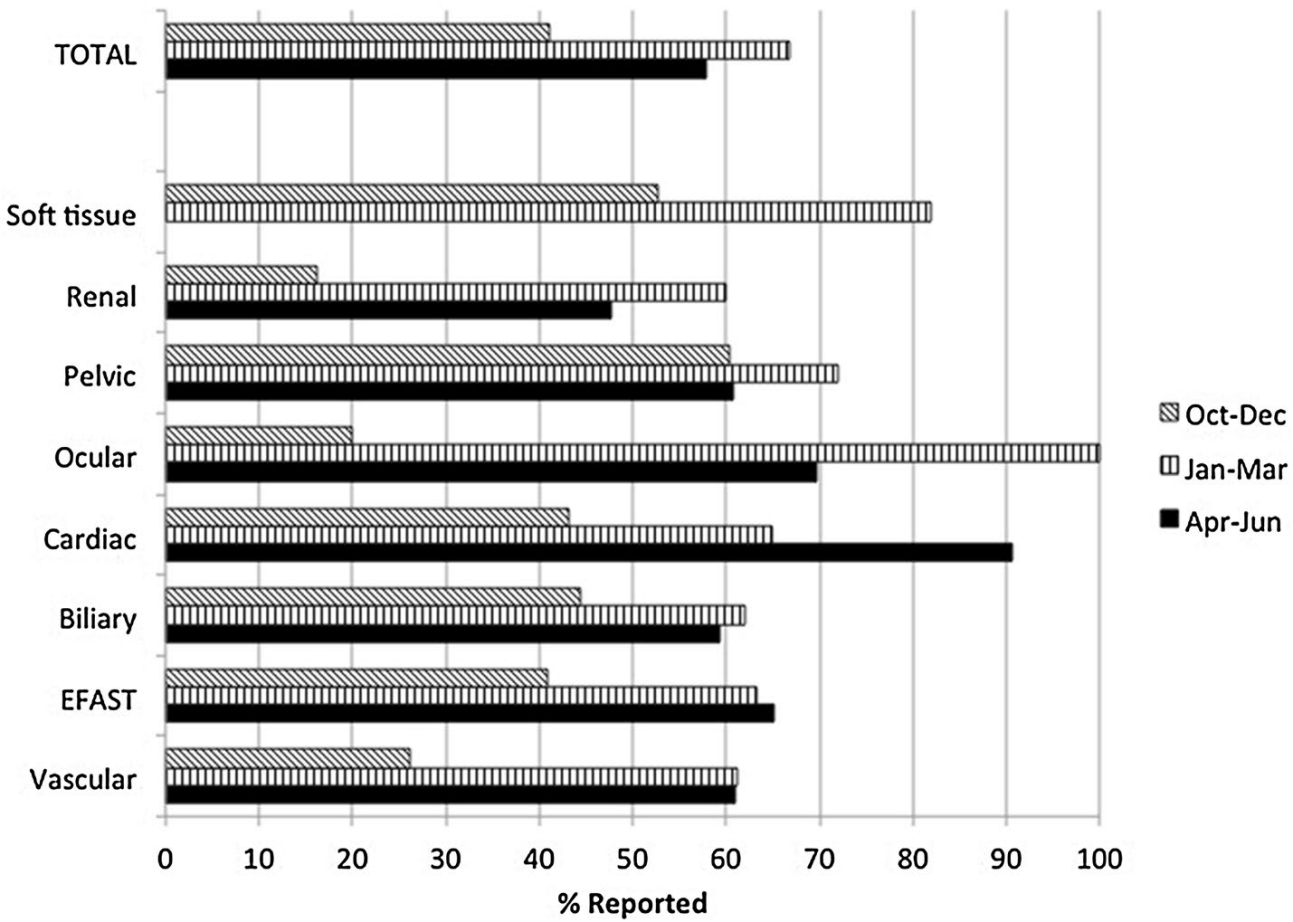
The percentage of scans reported to the medical coders in the pre- and two 3-month post-intervention periods. *X-axis* represents the percentage of POC u/s scans documented and reported for coding (*dark slanted hashed bar* pre-intervention data; *vertical hashed bar* first post-intervention data; *solid dark bar* second post-intervention period). *Y-axis* identifies the number of application specific and number of total POC u/s examinations

Figure 2 illustrates a comparison of the 3-month periods: pre-intervention, first quarter of the year and second quarter of the year.

**Fig. 3:**
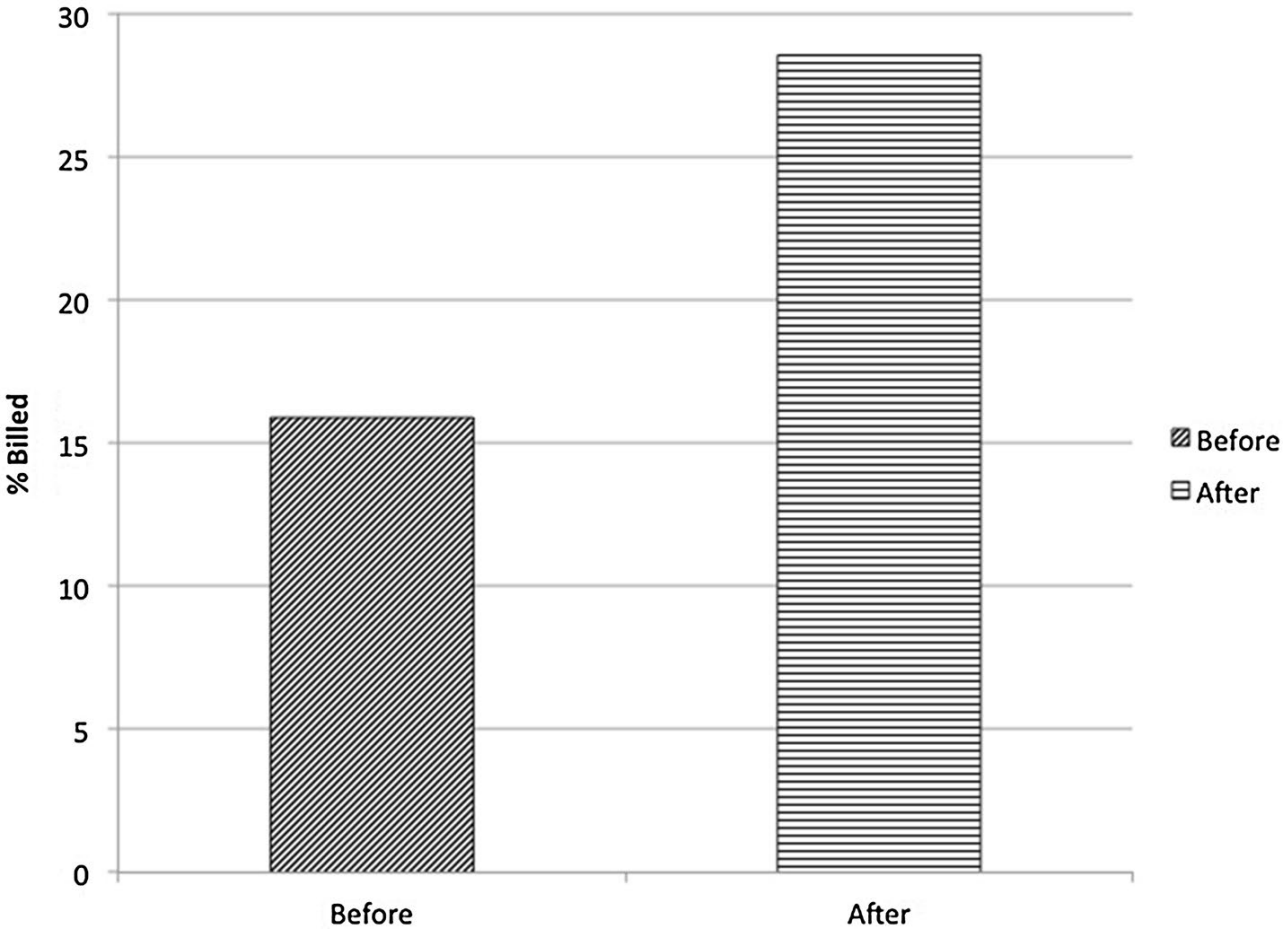
The change in the percentage of scans billed after being reported to the medical coders by the department pre- and post-task force intervention. *X-axis* illustrates pre- and post-intervention periods (*dark slanted hashed bar* pre-intervention data; *horizontal hashed bar* post-intervention data). *Y-axis* represents the percentage of examinations billed

For the majority of applications, health care provider documentation increased or remained stable. For the applications, where there appeared to be a decline in documentation, the numbers were too few to be statistically significant. Of the POC u/s studies coded and reported for reimbursement, 15.9 % were billed before intervention and 32 % were billed after intervention (*p* value: 0.000) (Fig. 3).

The number of ocular and thoracic POC u/s examinations submitted during the study period was too few to assess for a statistical change.

## Discussion

Encouraging behavioral changes in attending physicians is challenging. Attending physicians practicing in an academic environment represent a specific group of adult learners [7]. Grimshaw et al. suggest that active interventions, such as educational outreach and reminders to specific providers, are more effective than passive dissemination for changing provider behavior across a wide range of professional behavior change interventions. Additionally, multiple simultaneous interventions are more likely to be effective than any single intervention alone [7]. The formation of the ultrasound workflow solution task force was inspired by discussions with colleagues at other academic institutions and by review of relevant articles in the literature [8]. Many physicians felt the process was too time intensive, as it required logging into two software programs: the image database program and the EHR. We were able to utilize this feedback to simplify the workload of the physician as much as possible. For example, we had the auto logout time increased significantly to allow the physicians to remain logged into the database longer thus reducing the time required to document studies during their shifts. Interestingly, feedback from several physicians focused on medico-legal concerns as they were now placing a report of their US findings in the medical record prior to review by our ultrasound division. This concern was somewhat surprising to the department leadership as faculty had been credentialed to use US for clinical decision making for several years and they were already responsible for their interpretations. Through discussion at faculty meetings as well as individual conversations with concerned faculty members, we were able to allay this concern. It is likely that the intervention that is most effective once the workflow concerns were resolved was daily email feedback by our chairman to individuals who had not completed the workflow for billable studies. Faculty reported that while bothersome initially, the emails were highly effective in increasing compliance. We instituted systemic changes to improve the overall ease and acceptance of new compliance standards in ultrasound. We found that in the 3- and 6-month periods after the task force initiative, the mean number of POC u/s examinations documented and transferred for billing and coding significantly increased. This intervention is one that could potentially be replicated at other academic institutions with an organized POC u/s workflow.

While we have shown increased numbers of studies being documented and transferred in the short term, we cannot predict the long-term sustainability of our interventions.

### Limitations

One limitation of our study is the relatively brief post-intervention period. We may see deterioration in compliance over time. With multiple interventions employed at the same time, it is difficult to discern which form of feedback was most responsible for the change in behavior. A second limitation is the provider, who performed a “quick look” study for patient care without documentation or image archiving. This would not be accounted for in the denominator of the calculation. A third limitation is the Hawthorne or observer effect. Providers knew that the department chair was following the progress and completion of documentation so they have been more compliant for the period of study. In general, there were various time-intensive interventions implemented that changed practice in the short term; however, we do not know the long-term changes these will create. The interventions may not be feasible on a long-term basis. Finally, the study was performed at a single academic institution with an ultrasound division and fellowship. Some of the barriers and solutions may be specific to the institution and environment. A multi-centered study would be needed to analyze the specific effects in other practice environments.

## Conclusion

We are not aware of other studies looking at the formation of a workflow solution task force with the analysis of resultant behavioral changes in the successful documentation of POC u/s ultrasound examinations. The formation of a workflow solution task force positively affected emergency physician compliance with POC u/s documentation in the EHR for coding and billing. Ultimately, the formation of a task force may prove to be an efficacious method for providing feedback and incentive for behavioral change. A future study should seek to provide not only how the frequency of documentation of POC u/s changed, but also quantify the costs of the intervention and how that relates to the enhanced yields.
